# Effect of Curcumin-Nanoemulsion Associated with Photodynamic Therapy in Cervical Carcinoma Cell Lines

**DOI:** 10.1155/2018/4057959

**Published:** 2018-01-17

**Authors:** Renata Prandini Adum de Matos, Marilia Freitas Calmon, Camila Fernanda Amantino, Luisa Lina Villa, Fernando Lucas Primo, Antonio Claudio Tedesco, Paula Rahal

**Affiliations:** ^1^Institute of Biosciences, Humanities and Exact Sciences (IBILCE), Department of Biology, São Paulo State University (UNESP), Rua Cristóvão Colombo, 2265 Bairro Jardim Nazareth, 15054-010 São José do Rio Preto, SP, Brazil; ^2^Department of Chemistry, Center of Nanotechnology and Tissue Engineering-Photobiology and Photomedicine Research Group, Faculty of Philosophy, Sciences and Letters of Ribeirão Preto, University of São Paulo, Av. Bandeirantes 3900, Ribeirão Preto, SP 14040-901, Brazil; ^3^Center for Translational Research in Oncology, Instituto de Câncer do Estado de São Paulo (ICESP), Av. Dr. Arnaldo 251, 8° Andar, Bairro Cerqueira César, 01246-000 São Paulo, SP, Brazil; ^4^Faculty of Pharmaceutical Sciences of Araraquara, Department of Bioprocess and Biotechnology, São Paulo State University (UNESP), Rodovia Araraquara/Jaú, Km 01, s/n, Campos Ville, 14800-903 Araraquara, SP, Brazil; ^5^Faculdade de Medicina, Department of Radiology and Oncology, Universidade de São Paulo (USP), Av. Dr. Arnaldo 251, 8° Andar, Bairro Cerqueira César, 01246-000 São Paulo, SP, Brazil

## Abstract

Cervical cancer is the fourth cause of cancer death in women. Curcumin has antineoplastic properties. Furthermore, curcumin may be used as a photosensitizing agent in Photodynamic Therapy. This study aimed to investigate the effects of Photodynamic Therapy in cellular viability using curcumin-nanoemulsion as a photosensitizing drug in cervical carcinoma cell lines. The empty nanoemulsion presented very low cytotoxicity in all cell lines analyzed. Additionally, the incubation with curcumin-nanoemulsion at 20 *μ*M of curcumin showed more than 80% of cell viability for cell lines. Nanoemulsions were shown to be internalized inside cells by fluorescence microscopy and were observed in the intracellular environment for up to 36 hours after incubation with cell lines. In addition, after the Photodynamic Therapy we observed a high phototoxic effect of the curcumin-nanoemulsion with less than 5% of viable cells after irradiation. This was accompanied by an increase in caspase-3/caspase-7 activities after cell treatment with curcumin-nanoemulsion and Photodynamic Therapy, suggesting cell death by apoptosis. We conclude that the curcumin-nanoemulsion formulation behaves as a photosensitizing drug in Photodynamic Therapy and shows potential as an alternative treatment to cervical lesions using an endoscopic diode fiber laser setup for in situ activation or cavity activation using a diffuse fiber delivery system.

## 1. Introduction

Cervical cancer is the third most commonly diagnosed cancer worldwide and the fourth leading cause of cancer death in women [1]. In Brazil, there are an expected 16,340 new cases each year, with an estimated risk of 15.85 cases per 100,000 women for 2016 [2]. At present, the most effective treatment for cervical cancer is achieved through a combination of cisplatin-based chemotherapy with radiation [3]. However, the clinical use of this combination treatment is limited because of several serious side effects, such as nephrotoxicity, neurotoxicity, hematological toxicity, chemoresistance, and off-target damage to normal tissues [4]. In the early stages of the disease, this treatment regimen shows a patient survival rate of ~90%. However, the survival rates decrease to 17% for patients in advanced disease stages [4, 5] due to the presence of chemoradioresistant cells [6]. Therefore, ongoing efforts are necessary to develop new, effective therapeutic strategies to enhance chemotherapeutic efficacy and decrease these side effects, maximizing the local response and patient survival.

In the past two decades, the use of natural compounds with anticancer effects has attracted a great deal of attention as a strategy to overcome the low therapeutic index of conventional anticancer drugs. Chemopreventative phytochemicals target various intracellular signaling cascades to inhibit tumor promotion, proliferation, and progression [7]. Curcumin, a yellow pigment obtained from the rhizomes of* Curcuma longa* (Family: Zingiberaceae), is a major component of turmeric and is commonly used as a spice and food coloring agent [8]. Curcumin shows anticancer, antioxidant, and anti-inflammatory effects [9] and is known to induce apoptosis and block the progression of cervical cancer by downregulation of the TGF-*β* signaling pathway [10, 11]. For CasKi cells (cervical carcinoma cell line), curcumin inhibited the expression of VEGF, COX-2, and EGFR, which exhibited antitumor and antiangiogenesis effects [11]. Additionally, high concentrations of curcumin stimulated apoptotic cell death in several cervical carcinoma cell lines (SiHa, CasKi, and HeLa) by upregulation of Bax and AIF, downregulation of Bcl-xL and Bcl2, enhancement of cytochrome c release, and activation of caspase-3 and caspase-9 [12, 13]. Furthermore, curcumin has been applied in clinical trials for the treatment of pancreatic cancer [14], multiple myeloma [15, 16], Alzheimer's disease [17], and colorectal cancer [18].

Despite its potential therapeutic effects, the benefits of curcumin are limited due to its poor solubility, low absorption from the gut, rapid metabolism, and rapid systemic elimination [19, 20]. Therefore, new formulations based on nanoemulsions are currently being evaluated to increase the bioavailability and biological activity of curcumin [21–23]. Nanoemulsions are isotropic, thermodynamically stable, transparent (or translucent) systems of oil, water, surfactant, and cosurfactant with a droplet size usually within the range of 20–200 nm [24]. Nanoemulsions emerged as a promising tool for drug delivery owing to its long-term stability, ease of preparation, and high solubilization of drug molecules [25]. Additionally, many of the curcumin effects can be potentiated with the application of light. This approach, called photobiostimulation, has been used with success in clinical trials for early-stage skin cancer and other neoplastic diseases [26–30]. Curcumin can be used as a photosensitizer agent during Photodynamic Therapy (PDT), increasing the treatment efficiency and reducing the side effects.

PDT is a minimally invasive treatment strategy with low-toxicity [31–33]. Its components consist of a photosensitizing agent, a visible light source, and a molecular oxygen environment. It utilizes a molecular energy transfer from the photosensitizing agent, which absorbs visible light to form a newly excited species, to molecular oxygen, leading to cell and tissue toxicity via oxidative damage by reactive oxygen species (ROS) production [34, 35]. Photodynamic therapy selectively targets pathological cells and tissues through the local generation of highly toxic singlet oxygen and other toxic oxygen radicals (superoxide anion radical, hydrogen peroxide, hydroxyl radical, etc.), following activation of the photosensitizing agent by visible light that is within the therapeutic window (between 600 nm and 780 nm). Cell death is achieved through necrosis or apoptosis and is highly localized, causing little or no collateral damage [32]. The combination of curcumin with visible light results in significant phototoxicity in HeLa cells [36], L929 fibroblasts [37], and Lewis cells from lung carcinoma [38]. PDT has been indicated as a promising treatment for a wide range of cancers, such as cervical cancer and head and neck cancer [39–41]. Therefore, the aim of this study was to evaluate the effect of PDT and curcumin-nanoemulsion in cervical carcinoma cell lines.

## 2. Materials and Methods

### 2.1. Curcumin-Nanoemulsion: Preparation and Characterization

Curcumin-nanoemulsion (CNE) formulation was obtained, as described previously by Primo et al. [27], based on the interfacial prepolymer deposition and spontaneous nanoemulsification method [27]. Curcumin (Sigma-Aldrich, St. Louis, MO, USA) was entrapped in an oil phase at a final concentration of 0.1 mg/mL. The organic phase (acetone) was prepared, containing medium-chain-triglycerides and natural soy phospholipids (Lipoid S100, Lipid Co., Ribeirão Preto, SP, Brazil) at 55°C. Subsequently, this organic solution was added to the aqueous phase, containing an anionic surfactant, poloxamer 188 (Sigma-Aldrich Co., St. Louis, MO, USA). In the final step, the organic solvent was fully removed by rota-evaporation under reduced pressure at 60°C. All samples were prepared in aseptic conditions and in the absence of contaminants and chemicals interference. After preparation of the CNE, spectroscopic studies were performed at steady state through the spectrophotometer in the ultraviolet-visible (UV-Vis) range; absorption spectra were recorded on Lambda 20 Perkin Elmer double beam spectrophotometer (Perkin Elmer, Waltham, Massachusetts, USA) using 10 mm optical path length quartz cuvettes and 1200 nn·min^−1^ scanning speed. Absorbance was measured in the spectral range of 300 nm to 800 nm. Steady-state fluorescence spectra were acquired on Fluorolog Spex spectrofluorometer Jobin-Yvon/Horiba Instruments (Jobin-Yvon/Horiba Instruments, Chicago, Illinois, USA) using excitation and emission slits of 5 nm for each. Fluorescence emission was recorded in the 450 nm to 700 nm range, with the excitation wavelength fixed at 440 nm. Unless otherwise indicated, all measurements were carried out at 25°C [42].

To determine the particle size, the zeta potential was measured using the particle analyzer Zetasizer, model Nano ZS90 Malvern, operating at 633 nm and set to detect a scattering angle of 90° [27, 42].

### 2.2. Radiation Source

The radiation source used was a high potency LED apparatus, model Vet Light of DMC Enterprise (DMC Enterprise, São Carlos, São Paulo, Brazil), operating at 447 (±10) nm, with 420 mW of power at 23 mm and 2.52 W of total power, for 209 W/cm2 of irradiancy and 80 J/cm^2^ of fluency, set at 6.4 seconds/application.

### 2.3. Cell Culture

CasKi, SiHa, and HaCaT (spontaneously immortalized human keratinocytes) were obtained from ATCC and grown in cultured Dulbecco's Modified Eagle's Medium (DMEM, Gibco, Life Technologies, Carlsbad, California, USA) supplemented with 10% fetal bovine serum (FBS; Cultlab, Campinas, São Paulo, Brazil), 100 U/mL penicillin (Gibco, Life Technologies, Carlsbad, California, USA), and 100 mg/mL streptomycin (Gibco, Life Technologies, Carlsbad, California, USA). The cells were maintained in a humidified 5% CO_2_ incubator with a constant temperature of 37°C.

### 2.4. Cytotoxicity Studies

Cytotoxicity was assessed using the MTT classical assay, which is a nonradioactive, colorimetric assay. Media containing CNE, empty nanoemulsion (NE), and free curcumin at concentrations of 2 *μ*M, 5 *μ*M, 10 *μ*M, 20 *μ*M, or 40 *μ*M were added to each 96-well plate containing cells at the density of 2 × 10^4^. After 6, 12, and 24 hours of incubation, with the NE and CNE, MTT (1 mg/mL) (Sigma-Aldrich, St. Louis, Missouri, USA) was added to 100 *μ*L of medium in each well. Following 30 min of incubation at 37°C, the medium was removed and the formazan crystals were solubilized in 100 *μ*L of DMSO (Sigma-Aldrich, St. Louis, Missouri, USA). The absorbance of each well, which identifies the quantity of viable cells, was read at 570 nm on a microplate reader (Thermoplate model TP reader type B). The value at 690 nm (where no absorption was expected) was used as a reference. The optimal concentration to perform the following experiments was the concentration resulting in more than 80% cell viability without PDT.

### 2.5. Cellular Uptake of Curcumin

CasKi, SiHa, and HaCaT cells (3 × 10^5^/well) were plated in 6-well plates containing sterile round glass coverslips and stored under culture conditions. Cells were incubated with CNE at 20 *μ*M for CasKi, SiHa, and HaCaT cells. After 3, 6, 12, 24, 36, and 48 hours of incubation, living cells were viewed and photographed using a fluorescence microscope (Zeiss Axio Vert. A1) with a FITC filter (excitation spectra: 489 nm, blue laser, 415 ± 15 nm, FITC/GFP). Curcumin fluorescence could be detected by excitation at 410 nm and emission at 540 nm.

### 2.6. Cell Phototoxicity

All of the cell lines were seeded into 96-well plates at 2 × 10^4  ^cells/well and allowed to grow overnight to reach a confluent stage. On the day of the experiment, the cells were incubated for 3 h with CNE (20 *μ*M) or NE (20 *μ*M). After the incubation period, the culture medium was removed, cells were washed with PBS, and 150 *μ*L of fresh DMEM without phenol red was added to each well. Irradiation with laser was performed at 80 J/cm^2^ for 4 min. Cell viability was measured after 24 hours by the determination of mitochondrial activity by MTT assay.

Two different irradiation regimens were performed to analyze the efficiency of the PDT: only one irradiation dose at 80 J/cm^2^ performed after 3 hours of incubation; two irradiation doses (80 J/cm^2^ and 80 J/cm^2^) performed after 3 hours and after 36 hours (SiHa cells) or 48 hours (CasKi and HaCaT cells) of incubation for treatment with CNE. The time of the second irradiation for each cell line was the last experimental time where curcumin, as a photoactive compound, was detected intracellularly by fluorescence microscopy.

Controls were as follows: wells containing cells treated with photosensitizer but not exposed to light; wells containing cells without photosensitizer and without light; and wells containing cells without photosensitizer and exposed to light.

### 2.7. Cell Death upon Treatment

Caspase-3 and caspase-7 activities in CasKi, SiHa, and HaCaT cells were measured using a Caspase-Glo™ assay kit (Promega, Madison, WI, USA) after Photodynamic Therapy, using CNE as photosensitizer. Briefly, the proluminescent substrate, containing the DEVD, LETD, or LEHD (sequences are in a single-letter amino acid code), is cleaved by caspases. After caspase cleavage, a substrate for luciferase (aminoluciferin) is released; this results in the luciferase reaction and the production of the luminescent signal.

All cell lines were seeded into white-walled 96-well plates at 2 × 10^4^ cells/well and allowed to grow overnight. Cells were incubated with CNE (20 *μ*M) and NE, whereas the control group cells were incubated only with medium. After 3 hours the medium was removed, cells were washed with PBS, and 100 *μ*L of fresh DMEM without phenol red was added to each well and irradiated in the same conditions as item 2.6.

For the treatment with CNE, CasKi cells were incubated with fresh medium for 4.5 hours and SiHa and HaCaT cells for 8 hours after irradiation. The incubation time for each cell line was determined by an MTT assay performed after 3, 6, 9, 12, and 24 h. It was chosen according to the experimental time that produced ~50% of cell viability. After the incubation time, 100 *μ*L (1 : 1) of Caspase-Glo (Promega, Madison, WI, USA) reagent was added and incubated at room temperature for 1 h. The luminescence of each sample was measured in a plate-reading luminometer. The values were normalized with the control group value and expressed as percentages.

### 2.8. Statistical Methods

The statistical analyses were performed using GraphPad Prism 5.0 software (GraphPad Software, San Diego, CA, USA), and data were expressed as the mean plus standard deviation (SD). All data were tested for normality by Shapiro-Wilk test and the homoscedasticity by Levene test. Statistical analysis of nonparametric data was performed by Kruskal-Wallis test, and the parametric data were analyzed by one-way ANOVA with Tukey's test. The adopted confidence interval was 95%, with a significance level of *p* < 0.05.

## 3. Results

### 3.1. Particle Size and Zeta Potential Characterization of Curcumin-Nanoemulsion

The mean size distributions of the CNE and NE were determined by photon correlation spectroscopy using a Zetasizer Nano ZS of Malvern Instruments (Worcestershire, UK). This method allows the determination of the mean diameter of the particle (hydrodynamic radius), the polydispersity index (PdI), and the zeta potential of the particle population. The reported values were expressed as the mean ± standard deviation of at least three different batches of each formulation (Table 1). Values obtained are in agreement with previous reports of development of polymeric nanoemulsion and photosensitizers [27]. The encapsulation of curcumin into nanoemulsion does not change the particle size and zeta potential distribution, as shown in Table 1. The empty and loaded nanoemulsions have the same physical-chemistry profile, as expected for colloidal drug delivery systems [27]. Dynamic light scattering (DLS) analysis shows appropriate PdI values of <0.2 to curcumin-nanoemulsion, suggesting a monodisperse distribution in colloidal medium.

### 3.2. UV-Vis Absorption and Fluorescence Emission of Curcumin-Nanoemulsion

**Figure 1:**
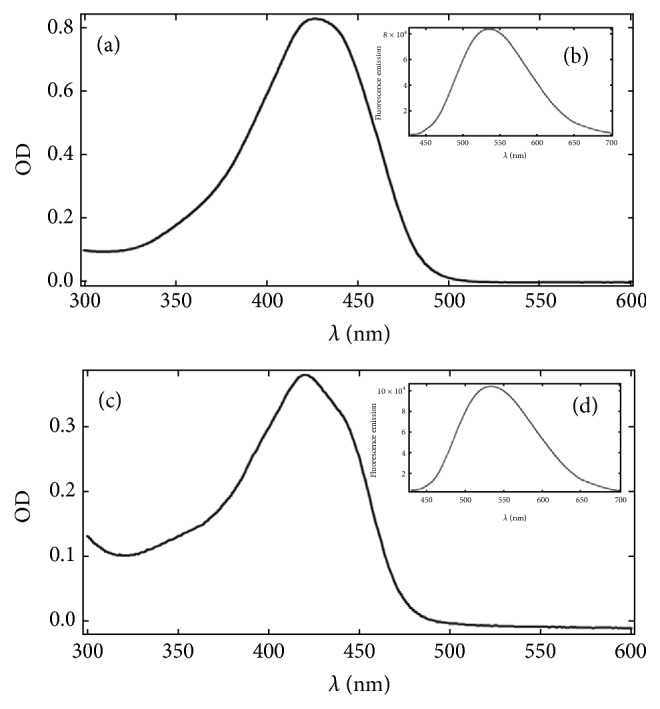
UV-visible absorption spectra for curcumin/methanol (a) and curcumin-nanoemulsion (c); fluorescence emission spectra at *λ* excitation = 415 nm with slits 5/5 nm for curcumin/methanol (b) and curcumin-nanoemulsion (d).

The results showed that the curcumin compound has a typical absorption band near the UV range, with a maximum peak at 415 nm. Fluorescence studies demonstrating the entrapment of curcumin into nanoemulsion produced a shift in the maximum emission wavelength from 540 to 490 nm. This result is expected for the colloidal environment and does not influence the photochemical and photobiological characteristics of the compound (Figure 1).

### 3.3. Effect of Curcumin on Cell Viability

**Figure 2:**
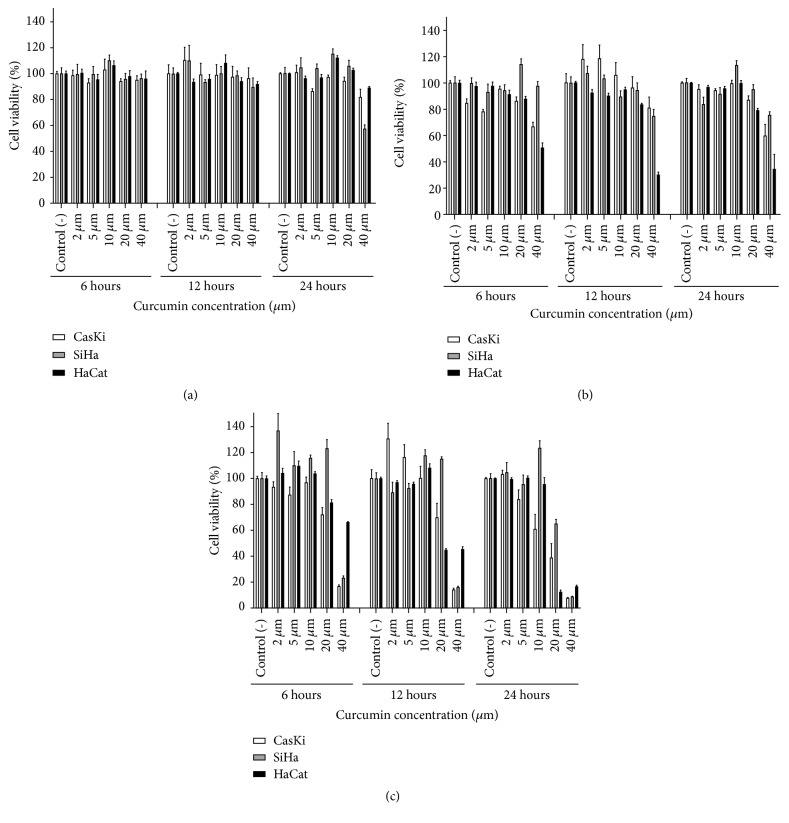
In vitro cytotoxicity of empty nanoemulsion, nanoemulsion-curcumin, and free curcumin. (a) In vitro cytotoxicity of empty nanoemulsion with equivalent doses of curcumin concentrations at 2 uM, 5 uM, 10 uM, 20 uM, and 40 uM in CasKi cells, SiHa cells, and HaCaT cells. (b) In vitro cytotoxicity of nanoemulsion-curcumin with curcumin concentrations at 2 uM, 5 uM, 10 uM, 20 uM, and 40 uM in CasKi cells, SiHa cells, and HaCaT cells. (c) In vitro cytotoxicity of free curcumin concentrations at 2 uM, 5 uM, 10 uM, 20 uM, and 40 uM in CasKi cells, SiHa cells, and HaCaT cells.

Curcumin cytotoxicity against CasKi, SiHa, and HaCaT cells was determined by MTT assay. NE did not show significant cytotoxicity, with most cell viability rates above 80%. (Figure 2(a)). On the other hand, cell viability was affected by curcumin in a dose-dependent manner.

When the three cell lines were treated with CNE at 20 *μ*M, the cell viability was above 80% for all analyzed times (Figure 2(b)). Therefore, we chose the 20 *μ*M concentration to perform additional experiments. However, we observed higher cytotoxicity for HaCaT cells than for CasKi and SiHa cells when the cells were incubated with the highest concentration of CNE (40 *μ*M).

All cell lines treated with free curcumin showed low viability at the same highest (40 *μ*M) concentration, with 7.8% of cell viability for CasKi cells, 8.6% for SiHa cells, and 16.5% for HaCaT cells, after 24 hours of incubation (Figure 2(c)). The concentration of free curcumin that maintained cellular viability above 80% during the three replicate experiments was 5 *μ*M for CasKi cells and 10 *μ*M for SiHa and HaCaT cells.

We demonstrated that CNE presented higher cell viability than free curcumin for the three cell lines in all experimental replicates that were analyzed. Therefore, the following experiments were performed only with 20 *μ*M CNE, as one of the characteristics of a good photosensitizer is low dark toxicity and strong photocytotoxicity [43].

### 3.4. Cellular Uptake of Curcumin

**Figure 3:**
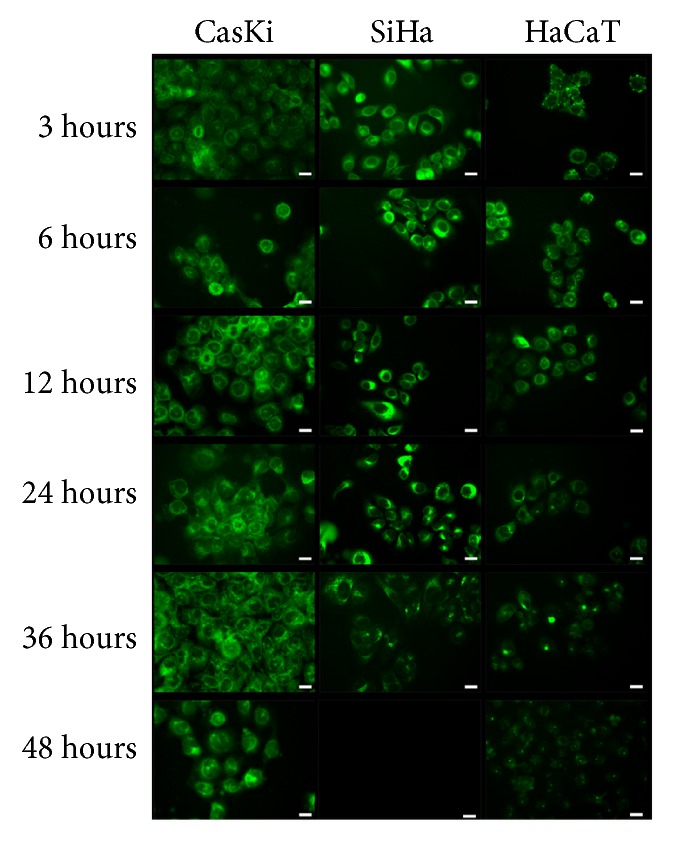
Fluorescence microscopy images of cells incubated with nanoemulsion-curcumin. The pictures were taken after 3, 6, 12, 24, 36, and 48 hours of incubation. Scale: 20 *µ*m.

Due to the natural fluorescent properties of curcumin, the cellular uptake of CNE was evaluated by fluorescence microscopy. The fluorescence microscopy images (Figure 3) showed that cells were able to internalize the CNE with preferential localization at cytoplasm, as observed in the intracellular environment for up to 36 hours after incubation in SiHa cells and for up to 48 hours in CasKi and HaCaT cells.

The fluorescent properties of curcumin also open the possibility of using the same active compound for diagnosis and/or treatment, following the theranostic protocol, as properties will be widely explored for this new generation of photoactive compounds.

### 3.5. Cell Phototoxicity

**Figure 4:**
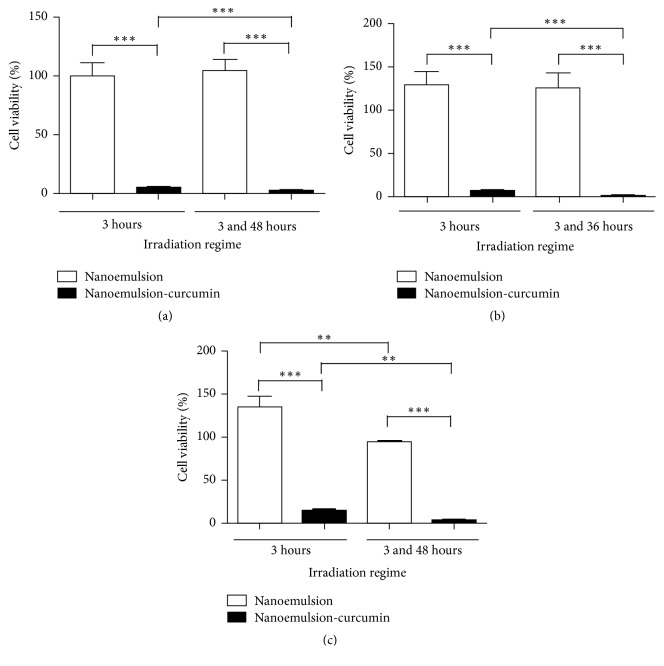
Phototoxicity assay in CasKi cells (a), SiHa cells (b), and HaCaT cells (c) under two different irradiation regimes. Percentage of cell viability performed with irradiation after 3 hours of incubation and irradiation carried out after 3 and 48 hours of incubation. ^*∗∗*^Significant difference, *p* < 0.001; ^*∗∗∗*^significant difference, *p* < 0.0001.

No decrease in cell viability was observed after incubation of the different cell lines with NE, showing that in the absence of the photosensitizing compound, the irradiation does not cause cytotoxicity (Figure 4). On the other hand, cells treated with CNE and irradiation showed a significant decrease in the cellular viability. After the treatment with one irradiation dose, we observed 5.4% of cellular viability for CasKi cells, 6.9% for SiHa cells, and 14.6% for HaCaT cells. After two irradiation doses, the cellular viability decreased to 2.8% in CasKi cells, 1.3% in SiHa cells, and 3.6% in HaCaT cells, and the difference in the cellular viability between those two treatments regimes was statistically significant (*p* < 0.0001 for CasKi and SiHa cells and *p* < 0.001 for HaCaT cells) (Figure 4).

### 3.6. Cell Death upon Treatment

**Figure 5:**
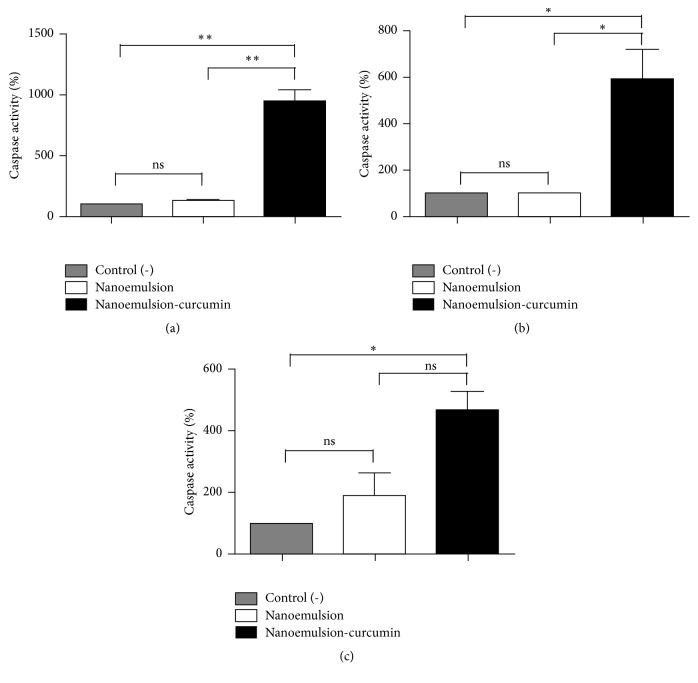
Enzymatic activity of caspase-3 and caspase-7 shown as a percentage in relation to the control samples (100%). (a) CasKi cells; (b) SiHa cells; (c) HaCaT cells. ^*∗*^Significant difference, *p* < 0.05; ^*∗∗*^significant difference, *p* < 0.001.

Caspase-3 and caspase-7 activities in CasKi, SiHa, and HaCaT cells were measured using a Caspase-Glo assay kit after PDT treatment with nanoemulsions. CasKi cells treated with CNE following one irradiation dose after 3 hours of incubation showed a significant increase of 847% (Figure 5(a)) in caspase activities in comparison with the control group. For SiHa and HaCaT cells, caspase activities increased by 492% (Figure 5(b)) and 366% (Figure 5(c)), respectively; these increased caspases activities in cells treated with CNE in comparison with control group were statistically significant for all cell lines (*p* < 0.001 for CasKi cells and *p* < 0.05 for SiHa cells and HaCaT cells). These results suggest that curcumin photoactivation triggers cell death by apoptosis. On the other hand, NE did not significantly increase caspase activity in comparison with control group, reinforcing the conclusion that the cell death by apoptosis results from curcumin photoactivation and was not influenced by nanoemulsion formulation.

## 4. Discussion

Curcumin is the main active compound of turmeric, a yellow compound isolated from* Curcuma longa* rhizomes [31]. Extensive research carried out during the last 30 years has shown that curcumin plays an important role in the prevention and treatment of various proinflammatory chronic diseases, including neurodegenerative, cardiovascular, pulmonary, metabolic, autoimmune, and malignant diseases [44–47]. Despite its safety and efficacy, free curcumin has not yet been approved as a therapeutic agent, mainly because of low bioavailability and low solubility in water [19]. However, several studies suggest that the problem of bioavailability can be solved through the association of curcumin with nanoformulations, such as liposomes, nanoparticles of different materials (PLGA, GMO, silica, gold, casein, and chitosan), complexes of phospholipids, hyaluronic acid, hydrogels, and nanoemulsions [48].

The nanoemulsion delivery system shows several advantages, including easy preparation, biocompatibility, small particle size (<200 nm), increased solubility, and protection against degradation of the chemical or biological drug [49]. Nanocurcumin has increased the therapeutic efficacy of free curcumin and has been used as an anticancer, neuroprotective, anti-inflammatory, and immunomodulatory agent [50].

Curcumin encapsulation within the nanoemulsion system enabled the increase of curcumin concentration without negatively affecting cell viability in our study, further corroborating other studies where curcumin nanoparticles have increased the cell viability in comparison with free curcumin in breast cancer cell lines [51], cervical cancer cell lines [52], and colorectal cancer stem cells [53]. Therefore, the use of nanotechnology allows for higher curcumin concentrations to be used without altering the cell viability.

The fluorescence microscopy images showed an efficient internalization of CNE formulation for all of the cell lines analyzed. As a lipophilic molecule, it is expected that free curcumin would localize to the membrane. We observed a wide distribution throughout the cytoplasm, indicating that curcumin encapsulation altered its lipophilic properties, facilitating its accumulation within the cell cytoplasm. On the other hand, other studies with free curcumin observed a high percentage of curcumin located in the cell membrane, followed by the cytoplasm, nucleus, and mitochondria in MCF7, NIH3T, and HeLa cells [54, 55].

Curcumin also presents photosensitizing and phytochemical properties, and accordingly it has been used successfully in Photodynamic Therapy to increase the treatment efficacy and decrease the side effects [56–58]. Due to the limitations of free curcumin for clinical trials (low solubility, fast degradation, and photochemical instability), a formulation capable of solubilizing and stabilizing the curcumin during the treatment is essential for photoactivity.

Nasopharyngeal carcinoma cells (NPC) treated with 20 *μ*M of free curcumin in the absence of light showed 70% cytotoxicity, whereas the same concentration associated with 300 J/cm^2^ of irradiation generated 95% phototoxicity [58]. Interestingly, we used the same concentration of CNE, presenting ~20% cytotoxicity in the absence of light, and irradiated the cells with 80 J/cm^2^, an energy almost four times smaller than that used in NPC, which produced 93.2% phototoxicity for CasKi cells, 83.16% for SiHa cells, and 96.31% for HaCaT cells and demonstrated great efficacy of our treatment system.

The photoactivation of nanoemulsion-curcumin generated a significant decrease in cell viability in all of the cell lines used in this study. Moreover, we observed an additional phototoxic effect when the cells were exposed to two irradiation doses (80 J/cm^2^ and 80 J/cm^2^) performed after 3 h and after 36 h (SiHa cells) or 48 h (CasKi and HaCaT cells) of incubation for treatment with CNE, which allowed the design of a new, more efficient treatment protocol.

The main advantage of PDT is that the damage is limited to the illuminated area without long-term, systemic side effects [59, 60]. Furthermore, there is no damage to normal structures, such as nerves, collagen fibers, and blood vessels [61]. Thus, despite the internalization of nanoemulsion-curcumin and phototoxic damage in HaCaT cells, the side effects of future treatment with our formulation can be minimized by directing the light source only to the precise anatomical sites that are affected.

One of the most interesting anticancer effects of curcumin is the induction of apoptosis. Apoptosis is a very complex, multistep, multipathway cell-death program that is genetically encoded in every cell of the body [18, 62, 63]. It can be initiated either through the activation of death receptors or the mitochondrial release of cytochrome c [20, 64]. Both events eventually lead to activation of caspase cascades, known as executioner caspases, such as caspase-3, caspase-6, and caspase-7 [65]. Activation of caspase-3 resulted in the apoptosis of cells, including cell shrinkage, membrane blebbing, and internucleosomal DNA fragmentation [66, 67]. As we observed an increase of caspase-3 and caspase-7 after the treatment with CNE and PDT, it is possible to conclude that the cell death observed is due to apoptosis triggered by the caspase pathway. However, it is not possible to determine if the activation of caspase-3 and caspase-7 that is observed occurs by either intrinsic or extrinsic pathways once they are executioner caspases [68]. The combination of PDT and curcumin in lung carcinoma cells and in laryngeal squamous cell carcinoma cells induced an upregulation of caspase-9 and caspase-3, suggesting that the intrinsic pathway (caspase 9) is involved in the curcumin-PDT-induced death of tumor cells [58, 69]. This may be present in some cells followed by a delayed engagement of the extrinsic pathway and activation of caspase-8, which can then directly cleave and activate caspase-3 and caspase-7, leading ultimately to apoptosis [65].

## 5. Conclusion

The nanoemulsion delivery system for curcumin developed in this study was internalized more efficiently than free curcumin for both cervical carcinoma cell lines (CasKi and SiHa) and in spontaneously immortalized human keratinocytes cell line (HaCaT). The nanoemulsions exhibited good cell viability since low cytotoxicity was seen for all of the cell lines that were analyzed.

The nanoemulsion-curcumin was capable of generating an efficient photodynamic response on carcinoma cervical cell lines, leading to death only in those cells that were irradiated. This protocol induced an increase in caspase enzymatic activities, suggesting that cell death occurs by apoptosis. We conclude that curcumin-nanoemulsion formulation behaves as a photosensitizing drug in Photodynamic Therapy and shows potential as an alternative treatment to cervical lesions, using an endoscopic diode fiber laser setup for in situ activation or cavity activation using a diffuse fiber delivery system.

## Figures and Tables

**Table 1:** Characterization of curcumin-nanoemulsion by dynamic light scattering technique.

Analyses	Nanoemulsion	Curcumin-nanoemulsion
Size (nm)	200 ± 9.9	199 ± 0.2
Zeta potential (mV)	−40.2	−46.3
Polydispersity index (PdI)	0.248	0.179
